# Lipase-Catalyzed Preparation and Optimization of Structured Phosphatidylcholine Containing Nervonic Acid

**DOI:** 10.3390/molecules29071539

**Published:** 2024-03-29

**Authors:** Xun Ang, Hong Chen, Jiqian Xiang, Fang Wei, Siew Young Quek

**Affiliations:** 1 Food Science Programme, School of Chemical Sciences, The University of Auckland, Auckland 1142, New Zealand; xang920@aucklanduni.ac.nz; 2 Riddet Institute, Centre for Research Excellence, Palmerston North 4474, New Zealand; 3 Institute of Oil Crops Research, Chinese Academy of Agricultural Sciences, The Key Lab for Biological Sciences of Oil Crops, Ministry of Agriculture—Hubei Key Laboratory of Lipid Chemistry and Nutrition, Wuhan 430062, China; chenhong@oilcrops.cn (H.C.); willasa@163.com (F.W.); 4 Enshi Autonomous Prefecture Academy of Agricultural Sciences, Enshi 445002, China; hmxjq@163.com

**Keywords:** nervonic acid, phosphatidylcholine, acidolysis, transesterification, phospholipase A_1_

## Abstract

This study investigated the incorporation of nervonic acid into the chemical structure of phosphatidylcholine via a lipase-catalyzed acidolysis reaction to obtain a functional phospholipid. Lipase immobilization was conducted, and Amberlite XAD7-HP was selected as a carrier to immobilize phospholipase A_1_ (PLA_1_) for subsequent experiments. The main acidolysis reaction parameters, including enzyme load, substrate ratio, temperature, and water content, were studied against the reaction time. The optimum reaction conditions obtained were enzyme load, 20%; reaction temperature, 55 °C; water content, 1%; and reaction time, 9 h. The maximum incorporation of nervonic acid into phosphatidylcholine was 48 mol%, with PC recovery at 61.6 mol%. The positional distribution of structured phosphatidylcholine shows that nervonic acid was found in the *sn-1* position due to enzyme specificity and in the *sn-2* position, possibly due to acyl migration.

## 1. Introduction

Nervonic acid (*cis*-tetracos-15-enoic acid; 24:1 Δ15, NA) is a long-chain monounsaturated fatty acid (FA) found in the white matter of animal brains and the myelin sheath of nerve fibers. NA is imperative for brain health and development as it helps to develop and maintain the brain by assisting in the biosynthesis and maintenance of nerve cell myelin [1]. NA is a natural component of human breast milk used as a nutritional supplement in infant formula to aid cerebral development in children. Additionally, supplementation for a pregnant or lactating woman can increase the NA proportion in their breast milk [2]. Therefore, there has been an interest in applying NA as a dietary treatment against neurological diseases as it supports the normal synthesis and function of myelin in brain and nerve tissues [1,2,3]. Research has been conducted using an in vitro human cell model to investigate the biological functions of NA [3]. These researchers reported that reduced levels of NA in blood or brain tissues are associated with metabolic syndrome and various neurological disorders, including Parkinson’s disease, schizophrenia, psychosis, attention deficit disorder, and multiple sclerosis [1,4,5]. The inclusion of NA in the diet has assisted in synthesizing myelin proteins, sphingomyelin and limiting inflammation that helps in the treatment of neurological diseases [3].

Phospholipids (PLs) are major constituents of biological membranes that play a vital role in the physiology and biochemistry of cells [6]. The amphiphilic properties of a PL create a lipid bilayer that facilitates the passage of FAs. These FAs pass through the intestinal walls and cell membrane assembly, increasing bioavailability [7]. Various research reported significantly higher bioavailability of FAs in PL form over triacylglycerol (TAG), ethyl esters, and free fatty acids (FFAs) [8,9]. The reason is that PLs undergo close to complete absorption (>90%) in the intestines via conversion to LPC and FFA [10]. In addition, a PL acts as an emulsifier to form a stable emulsion for absorption in the small intestine. However, TAG, monoacylglycerol, FFA, and ethyl esters were unable to leave the stomach for absorption as they could not form a stable emulsion [11]. Lemaitre-Delaunay, et al. [12] reported that docosahexaenoic acid (DHA) esterified to PL has a higher bioavailability for incorporation into erythrocytes in human adults than DHA esterified to TAG. Ramprasath, et al. [9] provided evidence that DHA esterified to PL was more effective than DHA esterified to TAG for increasing n-3 polyunsaturated fatty acid (PUFA) content, reducing the n-6: n-3 PUFA ratio, and improving the omega-3 index.

Structured phosphatidylcholines (SPCs) are phosphatidylcholines (PCs) with their structure modified using lipase as a catalyst through the incorporation or rearrangement of FAs on the glycerol backbone, aiming to alter and improve the physiochemical or nutritional properties. Lecitase Ultra (Phospholipase A_1_), Lipozyme TL IM, and Novozym 435 are commercially available phospholipases and lipases chosen for the alteration of PC. Their selection is based on their considerable potential for industrial scale-up in the production of structured lipids [13]. Additionally, Lecitase Ultra is the only commercial phospholipase A_1_ (PLA_1_) available and was initially designed for food application.

Previous researchers have prepared SPCs from medium-chain saturated FA (e.g., caprylic acid), conjugated linoleic acid, and n-3 PUFA from fish oil, including eicosapentaenoic acid and DHA substrates for incorporation into PCs [13]. Most studies have focused on utilizing DHA as a substrate for incorporation into PCs because of the beneficial effects of DHA on brain health. To the best of the authors’ knowledge, only one published research reported on preparing structured lipid using NA from *Acer truncatum* oil [4]. The research mainly focused on obtaining *trans*-free margarine fat containing only NA (1.28–2.41%) through chemical and enzymatic interesterification using *A. truncatum* oil, palm stearin, and palm kernel oil. The current research aimed to provide an effective method to prepare NA-enriched SPCs for potential use in food products. The scope of this research involved the immobilization of PLA_1_, followed by the screening of lipase, preparation and optimization of a lipase-catalyzed acidolysis reaction using PC and NA to obtain SPC.

## 2. Results and Discussion

### 2.1. Immobilization of PLA_1_

#### 2.1.1. Screening of Carrier

Immobilizing enzymes on a carrier can significantly improve its efficiency in the transesterification reaction [14]. The advantages associated with enzyme immobilization include increased catalytic activity, fewer effluent issues, ease of separation of enzyme and product, greater tolerance to pH, thermal stability, solvent, shearing forces, ease of recovery, and reusability [14]. A study by Li, et al. [15] reported that PLA_1_ in free form exhibited much lower catalytic activity. This is because PLA_1_ is an aqueous solution containing 56% water, which immensely accelerated the hydrolysis instead of the esterification. Another explanation could be the hydrophobic nature of FAs that may have led to insufficient contact with the PLA_1_ solution. In this experiment, PLA_1_ immobilization is conducted to increase the catalytic activity and for easy removal of the enzyme. Four carriers for PLA_1_ immobilization were investigated to screen and select the carrier with the highest specific activity and fixation level for subsequent optimization of the immobilization conditions. Fixation level is the % of enzyme adsorbed onto the carrier by weight. Therefore, ensuring that the fixation level is high is crucial to obtain a higher specific activity. In the preliminary experiments, Amberlite XAD-2 and Diaion HP-20 showed low fixation levels of less than 10% (supplementary materials). This was due to their hydrophobicity as they are found to give poor suspension properties in the enzyme solution. However, we found that pre-wetting hydrophobic carriers with ethanol before immobilization improved their suspension in the enzyme solution, which is consistent with the literature [15]. From Table 1, Amberlite XAD-7HP displayed the highest fixation level (94.16%) and specific activity. Supelite DAX-8 had similar specific activity to Amberlite XAD7-HP but with a lower fixation level (70.65%), indicating a lower load of PLA_1_ onto the carrier. Supelite DAX-8 has a favorable affinity with PLA_1_, leading to a high specific activity despite showing a lower fixation level than the Amberlite XAD7-HP. Nevertheless, the fixation level is an essential factor to consider. A higher fixation level indicates the higher adsorption of PLA1 onto the carrier, resulting in an increased mass transfer during the acidolysis reaction. Even though hydrolysis activity is not similar to esterification activity, it is frequently utilized as an indirect screening method for transesterification reactions. This is due to its simplicity, and the fact that hydrolysis of PC initiates before the esterification of FA [16,17]. Improving the fixation level is essential for economic feasibility due to the high cost of lipases. Based on the results, the Amberlite XAD-7HP was a suitable carrier and was selected for optimization of PLA_1_ immobilization.

#### 2.1.2. Specific Activity of Immobilized Enzyme

**Figure 1:**
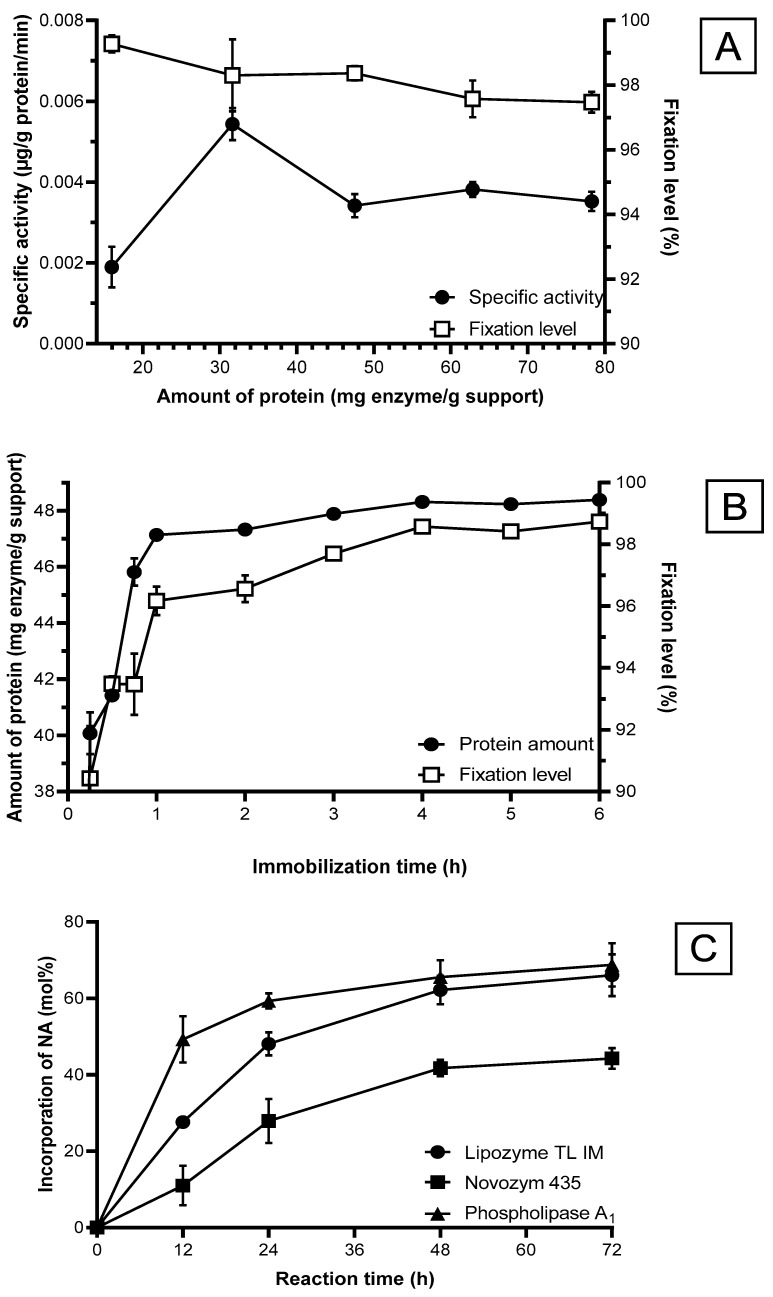
Optimization of PLA_1_ immobilization and lipase screening. (**A**) The relations of protein in immobilized enzyme (mg/g carrier) and the specific activity (µg/g protein/min) and fixation level (%) of PLA_1_ (hydrolysis reaction conditions: enzyme load 20%, temperature 50°C, reaction time 16 h); (**B**) Effect of reaction time on fixation level and the amount of protein adsorbed to Amberlite XAD-7HP (reaction conditions: Amberlite XAD-7HP 0.5 g, carrier/enzyme solution ratio (*w*/*v*) 1:4, temperature 30 °C, reaction time 1–6 h); (**C**) Effect of different lipases on the incorporation of NA into PC (reaction conditions: enzyme load 40%, reaction temperature 60 °C, substrate molar ratio 1:8 mol (PC:NA), water content 1%). Data shown are mean ± SD obtained from duplicate experiments.

The amount of PLA_1_ adsorbed onto Amberlite XAD7-HP was determined by measuring the amount of protein presence using the Bradford method [18]. The specific activity of the immobilized PLA_1_ was then determined (Figure 1A). The specific activity of the immobilized enzyme increased significantly from 0.00189 to 0.00543 µmol/g protein/min when the protein increased to 31.7 mg per g carrier (Figure 1A). As the protein increased above 47.5 mg/g, the specific activity of the enzyme decreased to 0.00341 µmol/g protein/min and then remained at around 0.00352 to 0.00382 µmol/g protein/min. The decrease in the specific activity is caused by a high amount of immobilized enzymes that increase steric restrictions and mass-transfer limitations through blockage or inhibition of the enzyme’s active sites [19]. Additionally, enzymes tend to pack beyond a monolayer on the surface of the carrier, causing restricted availability of surface area [19].

A high fixation level is essential for cost efficiency. However, high cost is the major concern of the food industry when considering the use of lipase and phospholipase for lipid modification. Considering the specific activity and the fixation level, 31.7 mg enzyme per g carrier was selected as the optimized ratio for the next section of the experiment.

#### 2.1.3. Immobilization Time

Figure 1B shows the effect of immobilization time on the fixation level and immobilized PLA_1_. The immobilized enzyme reached 47 mg per g carrier with a fixation level of over 96% in one hour. Previously published results obtained an optimized fixation level of immobilized PLA_1_ on DA-201 (82.5%) and Lewatit VP OC 1600 (79.5%) with an immobilization time of 7 h and 16 h, respectively [16,20]. Compared with the present study, the immobilization time of PLA_1_ was significantly reduced while attaining a significantly higher fixation level. This was achieved by pre-washing the Amberlite XAD-7HP with distilled water. As the carrier tends to adhere together, pre-washing helps separate the resin, thus increasing the surface area exposure for enzyme adsorption. Based on the results (Figure 1B), the immobilization time of 4 h is sufficient to obtain maximum enzyme adsorption onto the carrier (i.e., 48 mg/g) with a fixation level of over 98%.

### 2.2. Lipase Screening

The efficacy of the acidolysis reaction depends notably on the source of the enzyme. Other factors, including water activity, water content, pH, variation in affinity, relative enzyme specificity, substrates, and carriers for enzyme immobilization, can also affect the enzymes’ catalytic activity [21]. Therefore, screening for suitable lipase before selecting a suitable one is crucial to improve the optimization process of the acidolysis reaction [13]. Commercial immobilized lipases are preferred in most studies as optimizing the lipase immobilization steps are not required [22]. There is currently no commercial immobilized phospholipase; hence, PLA_1_ was immobilized on a carrier. Novozym 435 is a non-specific lipase immobilized via interfacial activation of lipase B from *C. antartica* on a resin, Lewatit VP OC 1600. Lipozyme TL IM is a *sn-1,3* specific lipase from *T. lanuginosus* on a non-compressible silica gel carrier, and Lecitase Ultra, a chimera produced by the fusion of the genes of the lipase from *T. lanuginosus* and the PLA_1_ from *F. oxysporum*, a *sn-1* specific phospholipase, were immobilized on Amberlite XAD-7HP via interfacial adsorption. The three lipases and phospholipase were studied for their incorporation of NA (Figure 1C). These lipases were selected based on their commercial/industrial application displayed in the current literature review associated with the structural modification of PLs [13]. In a review paper by Ang et al. [13], other commercial lipases have frequently been compared. However, their performance can vary due to differences in lipase specificity and experimental setup. Therefore, lipase screening remains essential, serving as the inaugural comparison between Lecitase Ultra, Novozym 435, and Lipozyme TL IM in terms of NA fatty acid incorporation.

Figure 1C displays the percentage of NA incorporation catalyzed by three lipases with the same reaction conditions. The sharpest increase in the incorporation of NA for all lipases ensues within the first 24 h. Subsequently, the curves for all lipases flatten out after 24 h as the acidolysis reaction reaches an equilibrium and the catalytic activity of lipase starts to decrease [23]. Novozym 435 displayed significantly lower incorporation of NA (40%) into PC after 72 h. Visual observation during the reaction revealed that the lipase adhered to the inner walls of the reaction tube during mixing, resulting in decreased mass transfer between the substrates. Lipozyme TL IM gradually increased NA incorporation into PC, reaching approximately 60%, slightly less NA incorporation than PLA_1_ after 72 h. PLA_1_ immobilized on Amberlite XAD7-HP achieved the highest NA (48%) incorporation into PC within the shortest time after 12 h. A possible explanation could be the superior affinity of phospholipase towards PLs. Therefore, PLA_1_ immobilized on Amberlite XAD7-HP was chosen as the lipase for subsequent acidolysis experiments based on the higher incorporation of NA in a shorter period.

### 2.3. Fatty acid Composition of SPC

Table 2 shows the FA composition of an unmodified PC and SPC. Linoleic acid (C18:2) is the most abundant FA (50.09%) in the PC, and no NA was present. After modification in the PC structure, the overall percentage of native FAs, especially linoleic acid (31.8%), decreased considerably, while NA increased to be the most abundant FA (46.72%). Previous research on SPC has reported a similar decrease in overall native FAs with a sharp increase in the newly incorporated FAs [20,24]. This was because PLA_1_ hydrolyses PC by cleaving at the *sn-1* position, releasing the native FA from the PC and incorporating NA into the PC via esterification [25].

### 2.4. Stereospecific Position of SPC

Table 2 shows the stereospecific positional distribution of FAs in the modified SPC. PLA_1_ is a *sn-1* specific phospholipase, which incorporated the majority of NA (63.5% ± 1.59) into the *sn-1* position of SPC. The *sn-2* position of SPC also contained NA (33.44 ± 2.73), possibly caused by acyl migration from *sn-2* to *sn-1* position or vice versa until a dynamic balance is reached. This phenomenon is often encountered in the selective synthesis of SPCs due to a change in the enzyme’s specificity or possibly from the enzyme immobilization procedure [26]. Another reason could be the reaction conditions that promoted acyl migration [14]. Acyl migration of NA into the *sn-2* position is not of major concern because the objective of the experiment was to obtain a high content of NA in a modified SPC. This may also give an insight into which position the FAs were esterified. It would also be beneficial to verify that NA incorporation does not happen in the absence of the lipase, since the acidic environment could promote low level of unspecific acidolysis. Additionally, FAs esterified in the *sn-2* position may be favorable as numerous studies have reported the potential enhancement of its digestion and intestinal absorption [27].

### 2.5. Parameters Affecting Acidolysis of PC and NA

The selection of the main parameters of acidolysis is crucial as it can significantly affect the incorporation efficiency of NA. The parameters applied in most studies (i.e., enzyme load, substrate ratio, time, temperature, and water content) are usually in the same range, depending on the substrates and lipases used [13]. Nonetheless, experimental verification of these parameters is still essential to improve the incorporation efficiency due to different experimental setups and affinities between substrates from each study. One-step acidolysis was selected for the lipase-catalyzed modification of PC with NA. This method allows hydrolysis and esterification reactions to occur in a single step, and sufficient product purity can be achieved without additional labor [23].

#### 2.5.1. Substrate Ratio

The substrate ratio is a parameter that determines the degree of FA incorporation into PC during the acidolysis reaction. Therefore, an appropriate substrate ratio is crucial to increase its incorporation efficiency. From our initial experiments, the substrate ratios 1:4, 1:6, and 1:8 mol:mol were used to evaluate the appropriate substrate ratio for subsequent experiments. After 24 h reaction, the results showed that a substrate ratio of 1:4 had the lowest NA incorporation efficiency (45.22%), followed by a ratio of 1:6 (NA incorporation of 56.04%) (Supplementary Figure S1). The lower incorporation efficiency was due to insufficient NA for incorporation into PC and mass transfer for homogenous mixing. To promote homogenous mixing and mass transfer, the submersion of lipase in a sufficient substrate is required [28]. The substrate ratio of 1:8 had the highest incorporation efficiency (59.25%) within the shortest time of 24 h (Supplementary Figure S1). When the substrate ratio increases, the increased FFA contributes to the solubility of PC, which subsequently promotes the mass transfer around the enzyme to promote the acidolysis reaction [20]. The substrate ratio of 1:8 was also applied in previous studies to synthesize SPC containing DHA [16,29].

The above results reflect the positive correlation between substrate ratio and FA incorporation into PC. However, it is crucial not to use an excessive substrate ratio. This is because the substrate inhibition effect, caused by reduced contact between substrate and enzyme, can result in hydrolysis inhibition in a solvent-free medium [28,29]. Using an excessive substrate ratio can also unnecessarily increase processing costs and cause product separation difficulty. Hence, the substrate ratio of 1:8 was selected for subsequent experiments.

#### 2.5.2. Enzyme Load

**Figure 2:**
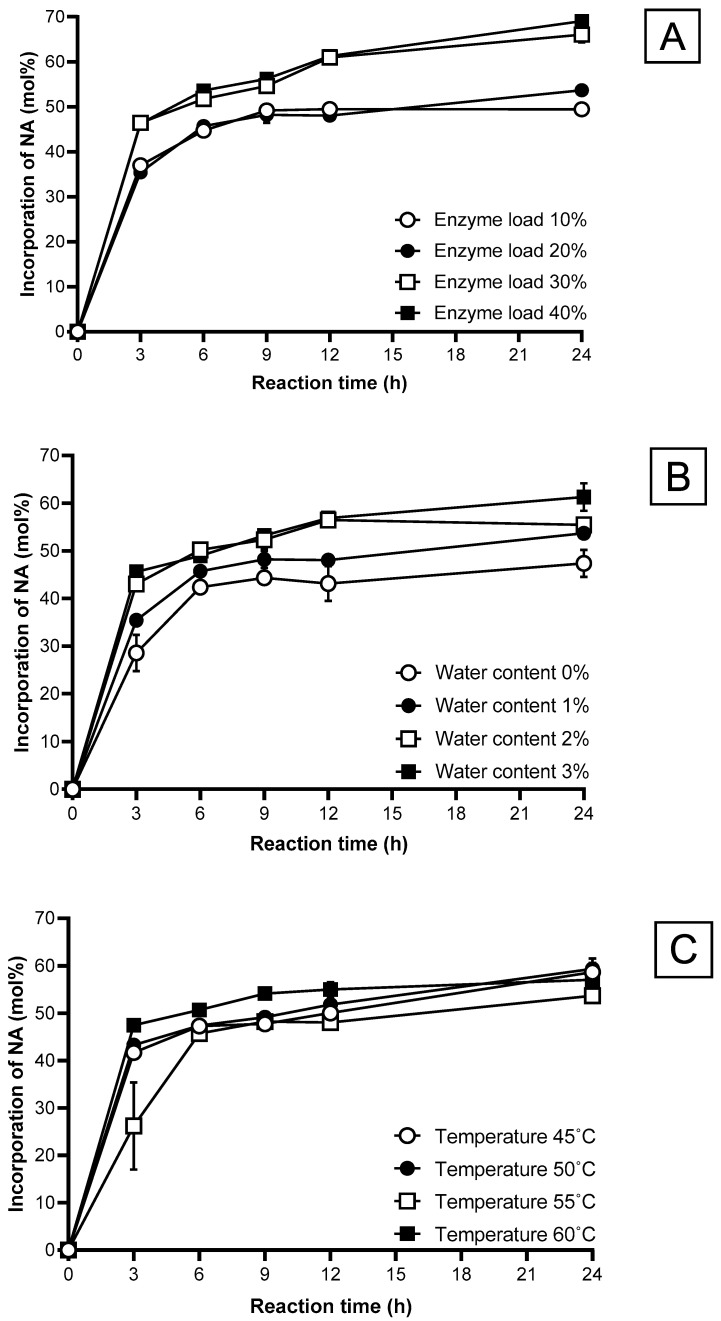
Incorporation of nervonic acid into phosphatidylcholine by acidolysis using phospholipase A_1_ for 24 h. (**A**) Effect of enzyme loading; (**B**) Effect of water content; (**C**) Effect of reaction temperature; substrate molar ratio was 1:8 (PC:NA) for the experiments. Data shown are mean ± SD obtained from duplicate experiments.

**Figure 3:**
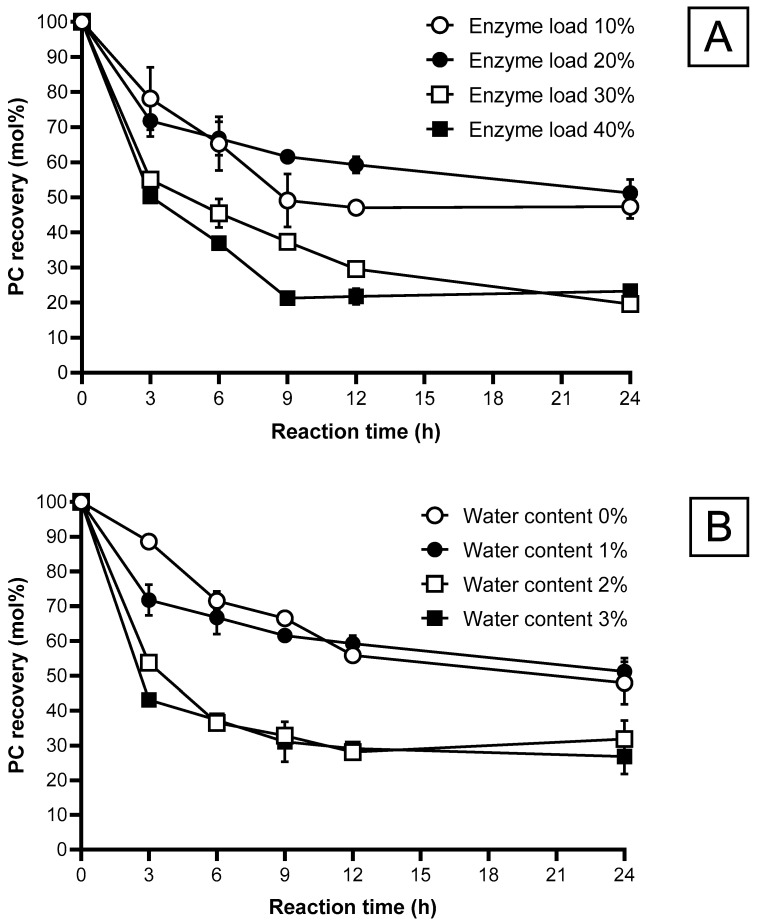

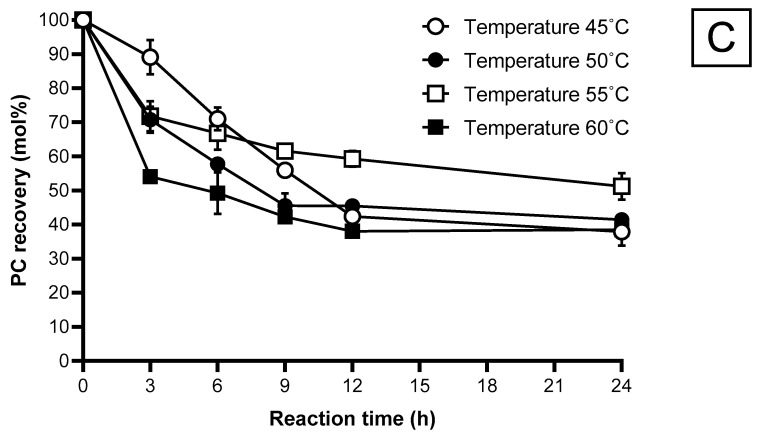
Phosphatidylcholine (PC) recovery of acidolysis using phospholipase A_1_ for 24 h. (**A**) Effect of enzyme loading; (**B**) Effect of water content; (**C**) Effect of reaction temperature; substrate molar ratio was 1:8 (PC:NA) for the experiments. Data shown are mean ± SD obtained from duplicate experiments.

Figure 2A and Figure 3A show the effect of enzyme load on NA incorporation and PC recovery. The highest increase in NA incorporation occurred in the first 3 h for all enzyme loads investigated (i.e., 10 to 40%). After 3 h, the NA incorporation rate increased very slowly. The enzyme load positively correlates with FA incorporation into PC, as shown in other studies [16,20]. This is because of the increased probability of collision with the enzyme and its substrate, subsequently enhancing the reaction rate [30]. A distinct difference was observed between the NA incorporation using an enzyme load of 10–20% (49–53% NA incorporation) and 30–40% (66–69% NA incorporation) at 24 h. However, no notable difference in NA incorporation was observed between 30 and 40% enzyme load due to the mass transfer limitation. An excessive amount of lipase may cause mass transfer limitation in the reaction, thus restricting the contact between substrates and lipase [28].

The highest decrease in PC recovery, 50.2 to 78%, was obtained at 3 h for all enzyme loads from 10 to 40% (Figure 3A). Then, the PC recovery decreased slowly, reaching 23.2 to 52%, depending on the enzyme load, at 24 h. The 10 and 20% enzyme loads significantly increased PC recovery of 47.32 to 51.22% compared to those obtained from the 30 and 40% enzyme loads. The results also showed a negative correlation between NA incorporation and PC recovery (Figure 2A and Figure 3A). This inverse relationship is unavoidable during acidolysis, mainly caused by a parallel hydrolysis reaction and acyl migration, increasing PC conversion to lysophosphatidylcholine (LPC) [31].

The 20% enzyme load displayed the highest PC recovery of 59.2%, alongside a relatively high NA incorporation of 48% at 12 h reaction time. Higher enzyme load (i.e., 30 and 40%) is unfavorable as PC recovery was much lower despite the higher NA incorporation. A high enzyme load can increase the processing costs and cause mixing difficulties, especially in a solvent-free system [28]. Thus, the enzyme load is compromised to balance the NA incorporation and PC recovery. Therefore, an enzyme load of 20% was chosen for subsequent acidolysis experiments, in agreement with previous studies that have also applied a similar enzyme load [16,21].

#### 2.5.3. Water Content

Water plays a vital role in the acidolysis and hydrolysis reactions as it can substantially control the reaction rate of lipase, PC recovery, and the incorporation efficiency of FAs. Figure 2B displays the effect of water contents of 0 to 3% on NA incorporation. It was observed that an increase in water content would increase the incorporation of NA. The presence of 1 to 2% water content significantly increased NA incorporation from 35.4% to 43% after 3 h. This is because a surplus of water content expedites the acidolysis reaction by promoting hydrolysis and aiding as a substrate for hydrolysis before the esterification of FAs [16]. The results obtained for water content agreed with the findings of a previous study [16,32]. Several studies reported very low FA incorporation when water was not added to the solvent-free system [29]. Enzyme does not function effectively when water activity is below its critical threshold in a reaction [16]. However, the NA incorporation at 0% water is only slightly lower than the value obtained at 1% water content. This somehow contradicts the argument that enzyme requires water to function effectively as a catalyst. Verdasco-Martín, et al. [24] reported that using a PC sample without dehydration severely affected reaction efficiency caused by undesired hydrolysis and strongly competes with the desired esterification of FAs. This could be caused by existing water in the substrates or water produced in the reaction adequate to maintain lipase activity in the reaction [33,34]. Li, et al. [35] reported that a hydrolysis reaction occurred even though the water addition was 0% wt which may be contributed by the inherent moisture of the immobilized PLA_1_ that results in the continued decrease in PC recovery.

A comparison of PC recovery between the results obtained from the 0–1% water content and 2–3% water content experiments demonstrate the significant negative effect of water on PC recovery. In the presence of 2 and 3% water during the reaction, the NA incorporation was 56%, with low PC recovery of 28.1 and 29.1%, respectively. This is because increasing water content can promote the esterification of FAs and hydrolysis side reactions to produce by-products (LPC). Over-hydrolysis can significantly decrease PC recovery and emulsion formation and pose complications in product recovery [16,29]. When selecting an appropriate parameter, it is imperative to consider the optimization of NA incorporation without the expense of PC recovery. In this regard, 1% of water content is a suitable parameter for the acidolysis reaction.

#### 2.5.4. Reaction Temperature

The selection of the appropriate reaction temperature is highly dependent on the melting points of the substrate (i.e., NA in the current study) and product [13]. The range of optimized temperature for the acidolysis reaction should be above the NA’s melting point (42–43 °C). This is crucial to ensure the substrates are dissolved adequately for efficient mixing in a solvent-free system. In addition to the substrate’s melting point, the lipase’s optimal temperature range is another important consideration. According to Novozymes, the optimum temperature range for the PLA_1_ is 35–60 °C; therefore, the selected temperature range for acidolysis is 45 to 60 °C.

Figure 2C shows the effect of temperature on NA incorporation. For the first 3 h, the NA incorporation rose sharply for all temperatures investigated (45, 50, 55, 60 °C). The reaction slows down after 3 h, similar to the other parameters. Temperature is commonly correlated positively to the reaction rate, according to Arrhenius’s law. Similar trends were observed in other studies, in which temperature increases led to an increase in FA incorporation [13,16]. This is because a rise in temperature increases the diffusion coefficient and the mass transfer rate. As a result, it enhances the contact between the substrate and the enzyme’s active site, resulting in higher incorporation levels of FA [20]. In this experiment, reaction temperature does not affect NA incorporation as much as the other parameters. A possible explanation could be that the temperature parameters selected were well within the optimal temperature range of the PLA_1_, according to Novozyme’s specification.

Figure 3C shows a significant decrease in the PC recovery for all temperatures. For the first 6 h, the PC recovery decreased at a higher rate when the reaction temperature increased. This was caused by increased hydrolysis, water activity, and acyl migration at high temperatures. Increased temperature can cause PC molecules to lose their stability, incorporating more FAs into the LPC rather than the PC [36,37]. High temperatures can also denature enzymes, causing reduced stability and catalytic activity. Unfortunately, hydrolysis is inevitable during an acidolysis reaction. Therefore, operating at a lower optimum temperature favors a lower rate of hydrolysis without influencing the primary reaction. At the lower reaction temperature (i.e., 45 °C), a higher PC recovery than other temperatures was observed initially due to a lower reaction rate. However, after 6 h, the PC recovery at 45 °C was lower than that obtained at 55 °C. This is unexpected as the lower hydrolysis rate at lower temperatures should translate to a slower decrease in PC recovery. A possible explanation is that the increased viscosity and decreased solubility of the substrates at lower temperatures could favor hydrolysis reaction over esterification [28]. Based on the results, 55 °C was selected as the optimal reaction temperature for the experiments because it gave the highest PC recovery (61.5%) while maintaining similar NA incorporation (48.2%) for a 9 h reaction period.

#### 2.5.5. Reaction Time

The selection of an optimal reaction time is imperative to acquire a maximal amount of NA incorporation and PC recovery. All parameters investigated, including enzyme load, reaction temperature, substrate ratio, and water content, enhanced the incorporation of NA over time from the increased contact between the substrates and lipase during the reaction. The reaction time course for each parameter of NA incorporation and PC recovery can be seen in Figure 2 and Figure 3. NA incorporation and PC recovery stagnate at 9 h or 12 h for most parameters. Any further increase in reaction time only results in a notable decrease in PC recovery without any substantial increase in NA incorporation. Therefore, 24 h was selected as the total duration for the acidolysis reaction for each parameter. Other studies chose 24 as the total acidolysis reaction time [13,16]. Even though prolonged reaction time is essential for a product’s formation, reaction time can be optimized to avoid over-hydrolysis and acyl migration to obtain a more economical operation cost.

### 2.6. Purification of Structured Phosphatidylcholine

**Figure 4:**
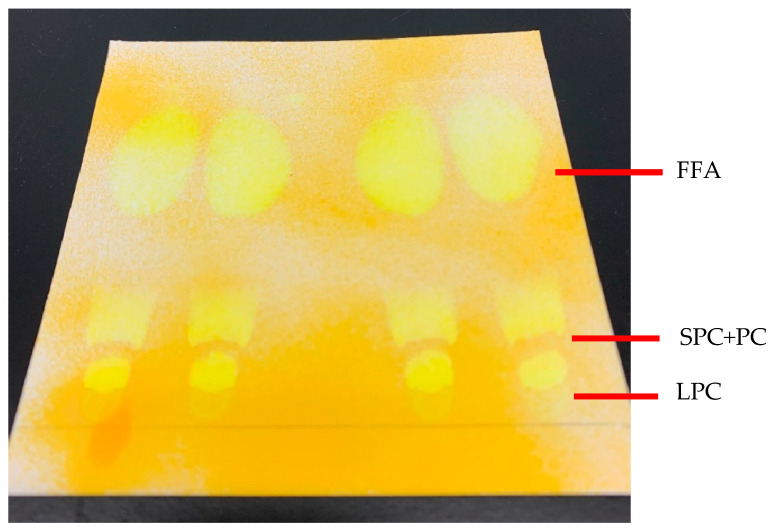
Band separation of the lipid classes on the TLC plate. 1st elution: FFA, 2nd elution: SPC+PC, 3rd elution: LPC.

Column chromatography successfully separated three lipid classes, FFA, SPC+PC and LPC, as shown in Figure 4. The ability to separate a large portion of lipids and its simplicity make column chromatography an efficient method to obtain pure SPC+PC from the lipid mixture consisting of PC, LPC and FFA. FFA was eluted first, followed by PC and LPC. Hence, the FFA had to be wholly eluted before the PC fraction was collected. The fractions for column chromatography were collected in a 1.5 mL centrifuge tube to monitor the time course of the lipid’s elution using TLC. The optimized parameters for SPC purification are 60 g of silica gel, 10 mL of final lipid mixture obtained from lipase-catalyzed acidolysis and the SPC+PC fraction collected between 1 h 15 min to 2 h 16 min. Before collecting the SPC+PC fraction, TLC was used to monitor the elution of the collected fractions to ensure no FFA and LPC were collected. Even though a high amount of SPC+PC was obtained, a small amount of FFA was still present in the purified SPC+PC, as the TLC plate showed a trace amount of FFA. This was similarly reported by Vikbjerg, et al. [38], who found low amounts of FFA but mentioned that it presented no issues due to their low concentration. The SPC samples were then collected and rotary evaporated to remove all traces of solvent. As the mobile phase for column chromatography contains water, the samples were freeze-dried to remove the remaining water.

**Figure 5:**
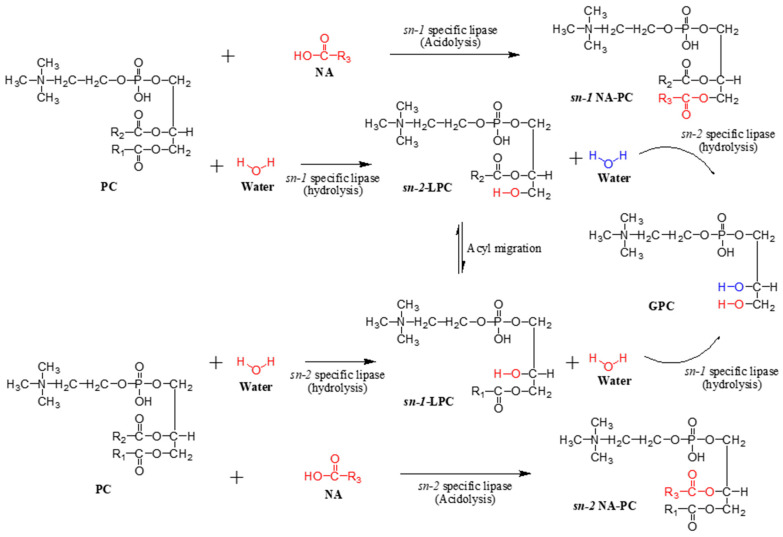
Summary reactions showing the lipase-catalyzed acidolysis of PC and NA, including hydrolysis of PC.

Figure 5 depicts the reaction’s mechanism involved in a lipase-catalyzed acidolysis reaction. Incorporating NA into the PC’s structure (*sn-1* and *2)* catalyzed by lipases is the primary acidolysis reaction. Hydrolysis side reaction coincides with esterification due to the presence of water. This induces the incorporation of water molecules into the PC structure to form LPC, depending on the lipase’s specificity [31]. The increased LPC formation from hydrolysis causes lipase to catalyze the incorporation of NA into the *sn-1* or *sn-2* position of LPC instead of PC [26]. Acyl migration can also ensue between *sn-1* and *sn-2* of LPC induced by further hydrolysis from excess water that leads to the formation of glycerophosphorylcholine (GPC) [26,37]. Over-hydrolysis induced GPC and LPC formation, leading to a significant decrease in PC recovery. Multiple studies have reported similar reaction mechanisms and reactant products that were obtained from transesterification reactions [27,35]. Due to the negative impact of hydrolysis on PC recovery, researchers have explored methods to improve PC recovery. This includes removing water by drying the PC substrate and utilizing a vacuum to suppress hydrolysis during acidolysis [16,20,24]. The literature underscores the benefits of utilizing solvents, such as improved mixing and easier enzyme separation from the product. However, drawbacks accompany solvent usage, including downstream processing challenges, environmental impact, and associated costs for industrial scale-up [13]. Conversely, solvent-free systems offer a safer, more cost-effective, and environmentally friendly alternative. Furthermore, they do not inhibit or denature the enzyme under certain conditions [37]. However, it is important to note that solvent-free systems may encounter issues like very low reaction rates, agitation problems, and difficulties in product separation.

## 3. Materials and Methods

### 3.1. Materials

Analytical standards Supelco 37-component fatty acid methyl ester (FAME mix), methyl heptadecanoate (C17:0), and methyl *cis*-15-tetracosenoate (C24:1) were purchased from Sigma-Aldrich (Castle Hill, Australia). Granulated soybean L-α-phosphatidylcholine PC95 (PC, purity > 95%) was obtained from Avanti Polar Lipids, Inc. (Alabaster, AL, USA). Lipases, including Lipozyme TL IM (from *Thermomyces lanuginose*, immobilized on silica gel carrier), Novozym 435 (from *Candida antartica*, immobilized on a microporous acrylic resin via interfacial activation), and Phospholipase A_1_ (Lecitase^®^ Ultra, from *T. lanuginose*/*Fusarium oxysporum*) were donated from Novozymes A/S (Bagsværd, Denmark). Amberlite XAD-7 HP, Amberlite XAD-2, Supelite DAX-8, and Diaion HP-20 were purchased from Sigma–Aldrich, Inc. (Castle Hill, Australia). Nervonic acid (98% purity) was purchased from Shenzhen Dieckmann Technology Development Co., Ltd. (Shenzhen, China). Chromatographic grade n-hexane, chloroform, and methanol were obtained from ECP Limited, Auckland, New Zealand.

### 3.2. Immobilization of Phospholipase A_1_ (PLA_1_)

Before immobilization, hydrophilic carriers Amberlite XAD7-HP and Diaion HP-20 were soaked in distilled water for 4 h and dried overnight. PLA_1_ was mixed with sodium phosphate buffer solution (50 mM, pH 7.5) in equal volumes of 5 mL. Enzyme suspension was stirred for 30 min and its soluble protein concentration was determined according to the Bradford method [18] using bovine serum albumin as the standard. Immobilization was conducted according to literature by Zhao, et al. [16] with slight modification. Carrier was added into the enzyme suspension at 1:10 ratio (*wt*/*v*) in a water bath with stirring at 200 rpm using a magnetic stirrer at 30 °C. The suspension was filtered after 16 h to recover the immobilized enzyme, followed by rinsing with 40 mL of sodium phosphate buffer. The immobilized enzyme was dried overnight at 45 °C in a vacuum oven and stored at 4 °C. The fixation level (wt %) and the amount of protein adsorbed to the carrier (mg/g) were estimated by subtracting the protein remaining in the enzyme suspension after immobilization compared with the initial protein concentration. The fixation level was calculated with the following equation:

(1) $$\mathrm{Fixation}\;\mathrm{level}\;(\%)=\frac{A-B}{A}\times 100$$

*A* = Initial protein amount in enzyme suspension (mg).

*B* = Final protein amount in enzyme suspension after immobilization (mg).

### 3.3. Determination of Enzyme Activity for Immobilized PLA_1_

The hydrolytic activity was used to determine the enzyme activity due to the esterification of FA which should be preceded by the hydrolysis of PC [16,20]. The activity of immobilized PLA_1_ was determined according to the method of Xi, et al. [20] with slight modifications by titrating the FFA released by the hydrolysis of PC emulsion with 20 mM NaOH. PC emulsion was prepared with an ultrasound homogenizer using 0.04 g/mL of PC95 in sodium phosphate buffer solution (50 mM, pH 7.5). The Scintillation vial (15 mL) containing PC solution was incubated in a water bath and stirred at 200 rpm with a magnetic stirrer at 50 °C. The hydrolysis reactions were initiated by the addition of immobilized PLA_1_. The specific activity was calculated below:

(2) $$\mathrm{Specific}\;\mathrm{activity}\;(µ\mathrm{mol}/g/\mathrm{protein}/\min)=\frac{x}{y\times z}$$

x = Hydrolyzed PC (µmol).

y = Amount of protein (g).

z = Initial reaction time (min).

### 3.4. Lipase-Catalyzed Acidolysis

An amount of 100 mg of PC95 and 370.9 mg of NA (1:8 mol PC/NA) were dissolved in a capped test tube under stirring at 100 rpm using a magnetic stirrer at 60 °C. Acidolysis reaction was initiated by adding lipase (10–40% enzyme load, based on total substrate weight) and water content (0–3% water content, based on total substrate weight). Screening of lipases was conducted with Lipozyme TL IM, Novozym 435, and PLA_1_ immobilized on Amberlite XAD-7HP. Reaction conditions used were optimized based on pre-screening studies (supplementary materials) using enzyme load (10–40%), reaction temperature (45–60 °C), reaction time (0–24 h for optimizing, 0–72 h for screening), substrate ratio (1:8 mol PC/mol NA) and water addition (0–3%). In duplicates, samples were extracted and analyzed at 3, 6, 9, 12, and 24 h. The addition of 1 mL solution of chloroform and methanol in a 2:1 ratio (*v*/*v*), respectively, was used for lipid extraction. The lipid solution was centrifuged to remove lipase, and the remaining sample was stored at −20 °C before analysis.

### 3.5. Analysis of Fatty Acid Composition by Gas Chromatography (GC)

FA analysis of NA crystals and positional analysis were determined according to Wei, et al. [39] with slight modifications. Sulphuric acid/methanol (2 mL, 5% *v*/*v*) and toluene (300 µL) was added with methyl heptadecanoate (C17:0) as an internal standard. The solution was mixed under reflux with nitrogen flushing for 1.5 h at 92–95 °C. Sodium chloride (2 mL, 0.9% *w*/*w*) and hexane (2 mL) were added to facilitate the extraction and phase separation.

Thin-layer chromatography (TLC) was used to analyze and separate the acidolysis reaction products. Samples collected at different time intervals were applied to TLC plates (Silicagel 60 F_254_ Merck Co., Darmstadt, Germany) and developed with chloroform/methanol/water (65:25:4 *v*/*v*). The bands corresponding to PC were scraped off the TLC plate and transferred into a test tube for FAME preparation [16]. SPC and PC FAME analyses were prepared using a previously published method by Hossen and Hernandez [21]. Methyl heptadecanoate (C17:0) was added as an internal standard.

Analysis of FAMEs was carried out on gas chromatography with a flame ionization detector (GC-FID) (Agilent 6890N, Palo Alto, CA, USA). The separation of FAMEs was performed on a capillary column (HP-FFAP, 30 m × 0.25 mm × 0.25 µm) purchased from Agilent Technologies (DKSH New Zealand Limited). Helium (purity ≥ 99.99%) was used as a carrier gas. The initial temperature of the oven was set at 150 °C. The temperature was programmed to 210 °C for 7 min at the rate of 10 °C/min. The temperature was further raised to 230 °C for 6 min at 20 °C/min. The injector and detector temperatures were 260 and 300 °C, respectively. FAMEs were identified based on the retention times of standards. The concentration of FAMEs was determined from the calibration curves of the measured peak area ratios. The initial and final PC (mol) amount was calculated from the total amount of FAMEs (mol) in each fraction. The incorporation of NA into PC and PC recovery were calculated below:

(3) $$\mathrm{Incorporation}\;\mathrm{of}\;\mathrm{NA}\;\mathrm{into}\;\mathrm{PC}\;(\%)=\frac{\mathrm{Incorporation}\;\mathrm{of}\;\mathrm{NA}\;\mathrm{into}\;\mathrm{PC}}{\mathrm{Incorporation}\;\mathrm{of}\;\mathrm{total}\;\mathrm{FA}\;\mathrm{in}\;\mathrm{PC}}\;\times \;100\%$$

(4) $$\mathrm{PC}\;\mathrm{recovery}\;(\%)=\frac{\mathrm{Mol}\;\mathrm{of}\;\mathrm{PC}\;\mathrm{after}\;\mathrm{reaction}}{\mathrm{Mol}\;\mathrm{of}\;\mathrm{initial}\;\mathrm{PC}}\;\times \;100\%$$

### 3.6. Analysis of Fatty Acids in sn-1 and sn-2 Position of Structured Phosphatidylcholine

The one-enzyme procedure method was modified from Kiełbowicz, et al. [40] to analyze the FA composition in the *sn-1* and *sn-2* position. Analysis of FA was conducted according to a method by Wei, et al. [39], albeit a modification using methanol instead of ethanol to convert FAs into FAMEs.

### 3.7. Purification of Structured Phosphatidylcholine

A chromatography column was used with 250 mL reservoir with an internal diameter of 20.0 mm and a length of 305 mm. The isolation of the NA-enriched SPC was conducted using column chromatography according to the method by Vikbjerg, et al. [38] with slight modification. The fractions of SPC+PC were collected, and the solvent was removed by rotary evaporation. The purified SPC+PC was pre-frozen at −80 °C for 12 h and subsequently lyophilised at −80 °C for 24 h with a freeze dryer (FreeZone 12 Plus, Labconco, MO, USA) to remove the remaining water.

## 4. Conclusions

SPC was successfully prepared by incorporating NA into PC via PLA_1_-catalyzed acidolysis. The main reaction conditions, including enzyme load, substrate ratio, reaction time, temperature, and water content, were optimized to obtain an appropriate balance between NA incorporation and PC recovery. Currently, minimal study has been conducted on the synthesis of SPC containing NA. Therefore, this research can bridge the knowledge gap to provide a reasonable basis for improving NA incorporation into PC in future work. In addition, a modified PC containing NA could potentially enhance the biological effects of NA for nutraceutical or medical applications. Further research could be performed to investigate the reusability of immobilized PLA_1_ and SPC’s bioactivity using animal models to better understand its biological mechanism.

## Figures and Tables

**Table 1:** The fixation level (%) and specific activity (µmol/g protein/min) of PLA_1_ immobilized on four different carriers.

Carrier	Fixation Level (%)	Specific Activity (µmol/g Protein/min)
Amberlite XAD-2	24.58 ± 4.2	9.4 × 10^−4^
Amberlite XAD-7HP	94.16 ± 0.31	2.9 × 10^−3^
Diaion HP-20	69.94 ± 2.22	2.2 × 10^−3^
Supelite DAX-8	70.65 ± 0.28	2.9 × 10^−3^

Fixation level % and specific activity were obtained from duplicate experiments. The values are presented as mean ± (SD) standard error.

**Table 2:** Fatty acid composition (mol %) of NA crystal, PC95, structured phosphatidylcholine (SPC) and positional distribution of *sn-1* and *-2* position of modified SPC.

Fatty Acid	NA	PC95	SPC	*sn-1*	*sn-2*
C16:0	-	24.19 ± 0.3	4.41 ± 0.71	3.28 ± 0.51	2.94 ± 0.61
C18:0	-	5.22 ± 0.04	0.61 ± 0.46	1.15 ± 0.19	2.59 ± 2.39
C18:1	-	9.27 ± 0.03	6.12 ± 0.28	3.93 ± 0.23	10.91 ± 2.92
C18:2	-	50.09 ± 0.25	31.8 ± 1.32	23.22 ± 1.15	44.18 ± 1.30
C18:3	-	11.4 ± 0.12	6.73 ± 0.30	2.27 ± 0.20	4.22 ± 0.92
C22:1	3.00 ± 0.18	-	1.09 ± 0.50	1.18 ± 0.22	0.89 ± 0.08
C24:0	4.64 ± 0.54	-	2.52 ± 0.19	1.48 ± 0.35	1.49 ± 0.35
C24:1	92.37 ± 0.71	-	46.72 ± 2.72	63.50 ± 1.59	33.44 ± 2.73

Fatty acid composition of PC95 and structured phosphatidylcholines data were mean ± SD obtained from duplicate experiments. Data for NA crystal, *sn-1* and *sn-2* position were mean ± SD obtained from triplicate experiments.

## Data Availability

The data presented in this study are available on request from the corresponding author.

## Supplementary Materials

The following supporting information can be downloaded at: https://www.mdpi.com/article/10.3390/molecules29071539/s1, Figure S1: Incorporation of nervonic acid into phosphatidylcholine by acidolysis using phospholipase A1 for 24 h. Substrate molar ratio used was 1:4, 1:6 and 1:8 (PC:NA mol:mol) for the experiments. Mean and standard deviation obtained from duplicate experiments. Figure S2: Fixation level (%) of phospholipase A1 on pre-wetted and non-pre-wetted carriers. Table S1: Oil extraction efficiency between enzymatic and solvent extraction.
