# Supplementary material for: Lipase-Catalyzed Preparation and Optimization of Structured Phosphatidylcholine Containing Nervonic Acid
Source: Molecules. 2024 Mar 29;29(7):1539. doi: 10.3390/molecules29071539 (PMC11013151; doi:10.3390/molecules29071539)
Supplement: Supplementary file 1 [file molecules-29-01539-s001.zip › molecules-2896315-supplementary.pdf]

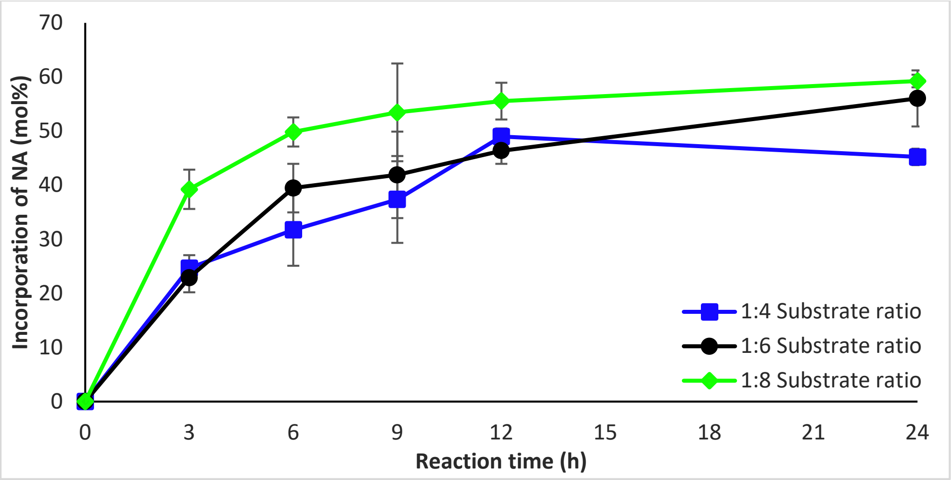

Figure S1: Incorporation of nervonic acid into phosphatidylcholine by acidolysis using phospholipase A_1 for 24 h. Substrate molar ratio used was 1:4, 1:6 and 1:8 (PC:NA mol:mol) for the experiments. Mean and standard deviation obtained from duplicate experiments.

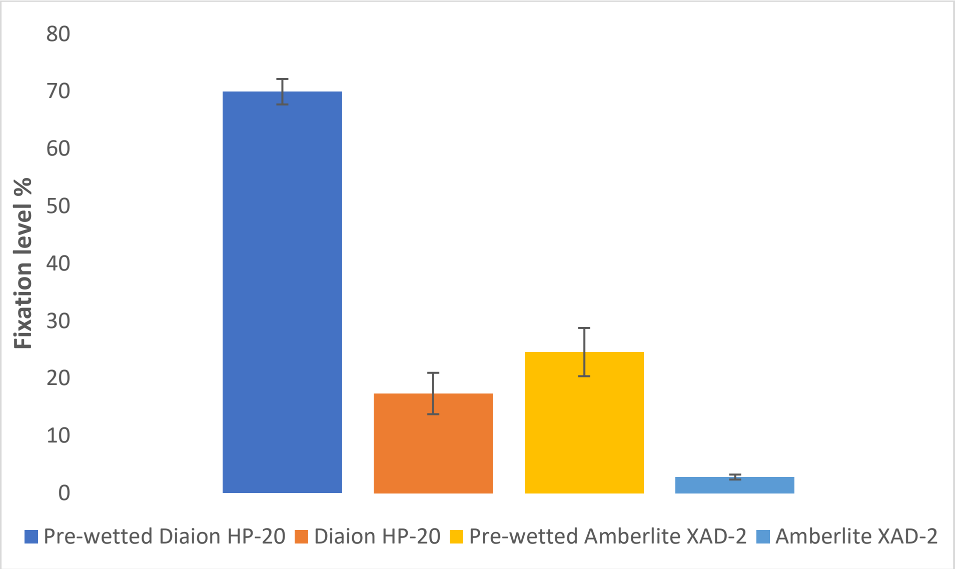

Figure S2: Fixation level (%) of phospholipase A_1 on pre-wetted and non-pre-wetted carriers.

Table S1: Oil extraction efficiency between enzymatic and solvent extraction

Method	Oil extracted (%)
Enzymatic extraction	33.56 ± 3.5
Solvent extraction	50.3 ± 4

Extraction were obtained from average of 4 samples.
